# Coagulation Profiles of Pulmonary Arterial Hypertension Patients, Assessed by Non-Conventional Hemostatic Tests and Markers of Platelet Activation and Endothelial Dysfunction

**DOI:** 10.3390/diagnostics10100758

**Published:** 2020-09-27

**Authors:** Eleni Vrigkou, Argyrios E. Tsantes, Petros Kopterides, Stylianos E. Orfanos, Apostolos Armaganidis, Eirini Maratou, Evdoxia Rapti, Athanasios Pappas, Andreas G. Tsantes, Iraklis Tsangaris

**Affiliations:** 1 Second Department of Critical Care Medicine, “Attikon” University Hospital, School of Medicine, National and Kapodistrian University of Athens, Rimini 1, 12462 Athens, Greece; elenivrigkou@gmail.com (E.V.); pkopterides@gmail.com (P.K.); stylianosorfanosuoa@gmail.com (S.E.O.); aarmag@med.uoa.gr (A.A.); pappasath@yahoo.gr (A.P.); 2 Laboratory of Hematology and Blood Bank Unit, “Attikon” University Hospital, Medical School, National and Kapodistrian University of Athens, Rimini 1, 12462 Athens, Greece; atsantes@yahoo.com (A.E.T.); evirap@yahoo.gr (E.R.); andreas.tsantes@yahoo.com (A.G.T.); 3 Laboratory of Clinical Biochemistry, “Attikon” University Hospital, Medical School, University of Athens, Rimini 1, 12462 Athens, Greece; maratou@hotmail.com

**Keywords:** bloodcoagulation disorders, platelet aggregation, p-selectin, pulmonary arterial hypertension, serotonin, thrombin, thromboxane A2, von Willebrand

## Abstract

Many pathophysiologic processes of pulmonary arterial hypertension (PAH), namely, excess vasoconstriction, vascular remodeling and in situ thrombosis, involve the coagulation cascade, and more specifically, platelets. The aim of this study was to globally assess coagulation processes in PAH, by using non-conventional hemostatic tests, along with markers of platelet activation and endothelial dysfunction. We studied 44 new PAH patients (22 with idiopathic PAH and 22 with connective tissue disease) and 25 healthy controls. The following tests were performed: platelet function analyzer-100 (PFA-100), light transmission aggregometry (LTA), rotational thromboelastometry (ROTEM), endogenous thrombin potential (ETP), serotonin, thromboxane A2 and p-selectin plasma levels, and von Willebrand antigen (VWF:Ag) and activity (VWF:Ac). Our results showed that PAH patients had diminished platelet aggregation, presence of disaggregation, defective initiation of the clotting process and clot propagation, and diminished thrombin formation capacity. Serotonin, thromboxane A2 and p-selectin levels were increased, and VWF:Ag and VWF:Ac decreased in the same population. The results of this study suggest that the platelets of PAH patients are activated and present functional abnormalities. The procoagulant activity, in general, appears to be impaired probably due to a sustained and prolonged activation of the procoagulant processes. Larger observational studies are warranted to confirm these laboratory findings.

## 1. Introduction

Pulmonary arterial hypertension (PAH) is a chronic and progressive disorder where structural and molecular changes of the pulmonary circulation result in elevated pulmonary vascular resistance, right-sided heart failure and potentially death [1]. Τhe most crucial pathophysiological characteristics of PAH involve endothelial dysfunction, excess vasoconstriction, vascular remodeling and in situ thrombosis [2]. Despite the substantial progress that has been made in the last decades, essential pathophysiological processes still remain elusive [3]. One of the most controversial areas on the subject refers to the role of platelets, and coagulation in general, in the emergence and development of PAH [4].

Coagulation processes are involved in most of the major pathophysiological pathways of PAH, either directly (e.g., thrombus formationandthrombotic arteriopathy) or indirectly (through the production and release of vasoactive substances, inflammatory cytokines, and mitogenic and growth factors) [4]. Evidence of platelet function abnormalities and dysregulation of the coagulation cascade have been found in PAH patients [5]. The frequently contradictory reports found in the current literature, however, along with the relatively limited number of relevant studies, make it difficult for the complete nature of hemostatic abnormalities in the PAH setting to be adequately assessed [5].For example, even though classic studies in the PAH setting have shown evidence of vascular thrombotic lesions and in situ thrombosis in the small caliber peripheral pulmonary vessels in post-mortem evaluations in patients suffering from idiopathic pulmonary arterial hypertension (iPAH), some more recent studies were not able to provide evidence of increased thrombogenesis in the PAH population, while othersdid [5,6,7].Studies assessing markers of fibrinolysis have also rendered inconsistent results with some studies showing increased and others decreased fibrinolysis [5,7]. Furthermore, it has been observed that PAH patients frequently present with thrombocytopenia, the etiology of which has not yet been identified [8].

In a previous study [9], we demonstrated that platelet function, thrombin generation, and clot initiation and propagation are impaired in newly diagnosed PAH patients. The aim of the present study was to validate our previous results and to further elucidate the underlying mechanisms of the hemostatic abnormalities observed in PAH patients, by assessing markers of platelet activation and endothelial dysfunction. For this purpose, serotonin and thromboxane A2 (TXA2) plasma levels were assessed as markers of platelet activation [7,10], von Willebrand antigen (VWF:Ag) and activity (VWF:Ac) as markers of endothelial dysfunction [11] and soluble p-selectin as a marker of both platelet activation and endothelial injury [11].

## 2. Materials and Methods

### 2.1. Study Population

We recruited 44 consecutive subjects with PAH, at the time of their diagnosis: 22 with connective tissue disease (CTD-PAH), more specifically systemic sclerosis, and 22 with idiopathic PAH (iPAH). None of the patients participated in our previous study [9]. Diagnosis was made according to current European Society of Cardiology and European Respiratory Society (ESC/ERS) guidelines [1]. Blood samples were collected directly from the patients’ pulmonary artery during the initial right-heart catheterization. The control group consisted of 25 healthy volunteers and was comparable in age and sex with the patient group. Blood samples from the control group were drawn from an antecubital vein. The study’s exclusion criteria were: history of coagulation disorders, hepatic, renal and thyroid insufficiency, malignancy, active infections, use of antiplatelet or anticoagulation therapy, and use of non-steroidal anti-inflammatory drugs (NSAIDs) for 3 weeks prior to study inclusion. The study was approved by the review board of ‘Attikon’ University Hospital (ΕΒD410/17-9-14) and all procedures were performed in accordance with the amended Declaration of Helsinki.

### 2.2. Global Evaluation of Hemostasis

Hemostasis was evaluated by 4 different laboratory methods, all of which were thoroughly analyzed in our previous paper [9]. In the following sections, these techniques are briefly explained.

1. Primary hemostasis was assessed by the platelet function analyzer-100 (PFA-100) (Dade Behring, Marburg, Germany). A total of 0.8 mL of whole blood collected in 3.8% trisodium citrate were placed in the sample reservoir of a collagen/epinephrine cartridge (Dade Behring, Marburg, Germany), within 3 h of blood collection, and then processed by the analyzer. In this test, shear stress and epinephrine (EPI) are used to activate platelets. The platelets then aggregate and, subsequently, close an aperture in the cartridge where the test is performed [12]. The measured parameter is the time needed for the aperture to close (closure time (CEPI CT)).

2. Platelet aggregation was evaluated using the Light Transmission Aggregometry (LTA) (Biodata-PAP-4 instrument, Bio/Data Corp., Horsham PA, USA), a gold-standard measure for assessing platelet function [13]. This method estimates light transmission changes through the different stages of platelet aggregation [13]. This test was performed within 3 h of blood collection. A whole blood sample collected in 3.8% trisodium citrate was centrifuged at 200× *g* for 10 min at room temperature, providing us with the platelet-rich plasma. The platelet-poor plasma, used to set the 100% line, was prepared by recentrifuging the remaining specimen at 2000× *g* for 15 min. The test was performed by adding 0.05 mL ADP 2.0 × 10^−5^ M or 0.05 mL Epi 1.0 × 10^−4^ M (Bio/Data Corp., Horsham, PA, USA) in 0.45 mL of platelet-rich plasma and subsequently processed by the aggregometer. The results are calculated as % maximal aggregation. The disaggregation measurement was provided in cases where the late aggregation value did not correspond to the peak value.

3. The quality of hemostasis was evaluated by assessing the viscoelastic characteristics of blood clots, using the rotational thromboelastometry (ROTEM) (Tem Innovations GmbH, Munich, Germany) [14]. We performed the non-activated TEM (NATEM) assay, were 20 μL of CaCl2 0.2 mol/L (Tem Innovations GmbH, Munich, Germany) were added in 300 μL of a whole blood sample collected in 3.8% trisodium citrate, within 3 h of blood collection. ROTEM’s measured parameters are summarized in Table 1.

4. We used endogenous thrombin potential (ETP) (INNOVANCE^®^, Siemens Healthcare Diagnostics, Marburg, Germany) to evaluate the amount and time course of thrombin generation [15]. Blood samples were collected on 3.8% trisodium citrate anticoagulant and immediately centrifuged at 2500× *g* for 20 min. The supernatant plasma was removed, snap frozen in small portions and stored at −20 °C until the assays were performed. ETP’s result parameters are depicted in Table 2.

### 2.3. Markers of Platelet and Endothelial Dysfunction

Plasma samples for these assays were obtained as for the ETP testing. TXA2, serotonin and p-selectin plasma levels were quantified by commercially available ELISA kits. TXA2 kit (American Research Products, Inc., Waltham, MA, USA) has a minimum detection limit of 5.62 pg/mL, and inter-assay and intra-assay precision of less than 12%. Serotonin kit (Enzo Life Sciences, New York, NY, USA) has a minimum detection limit of 0.293 ng/mL, and inter-assay and intra-assay precision of less than 13%. The P-selectin kit (Young in Frontier Co, Ltd., Seoul, Korea) has a minimum detection limit of 5 pg/mL.

VWF:Ag and VWF:Acwere determined by an automated latex enhanced immunoassay (HaemosIL™ assay, Instrumentation Laboratory Company, Lexington, KY, USA) on IL (Instrumentation Laboratory) coagulation systems (ACL TOP). VWF:Ac was assessed by measuring the variations in turbidity generated by the agglutination of the latex reagent [16]. An anti-VWF monoclonal antibody adsorbed onto the latex reagent reacts with the VWF of the blood sample. Agglutination is proportional to VWF:Ac and is calculated by the diminished light transmission caused by the aggregates. Results are presented as percentageof normality.

### 2.4. Conventional Coagulation Tests

Conventional coagulation tests were performed in all study participants. Complete blood counts were measured on a Sysmex XE-2100 analyzer (Roche, Chicago, IL, USA). aPTT, PT, INR, fibrinogen and D-dimers were determined on a BCS^®^ XP System Hemostasis analyzer (Siemens Healthcare Diagnostics, Marburg, Germany).More specifically, Pathromtin SL (Siemens Healthcare Diagnostics, Marburg, Germany) was used to assess aPTT and Thromborel S Reagent for PT calculation. Determination of fibrinogen concentrations were made performing a modification of the Clauss method with Fibrinogen Multifibren U reagent (Siemens Healthcare Diagnostics, Marburg, Germany). D-dimers were assessed by the INNOVANCE D-Dimer assay (Siemens Healthcare Diagnostics, Marburg, Germany), a particle-enhanced immunoturbidimetric method.

### 2.5. Statistical Analysis

Summary statistics are depicted as means ± standard deviations (SD), medians and interquartile ranges (IQR), or percentages. Statistical evaluations were performed using non-parametric methods (Two sample Wilcoxon rank-sum (Mann–Whitney) test). Probability levels lower than 0.05 were considered as statistically significant. Statistical tests were 2-sided. Stata software (Stata Corp., College Station, Texas, TX, USA) was used for all statistical analysis.

## 3. Results

We present the demographic data and laboratory findings of all study participants in Table 3. In the same table, we include the hemodynamic, laboratory and clinical features of PAH patients.

### 3.1. Global Evaluation of Hemostasis

The results of the PFA-100, LTA, ROTEM and ETP assays are presented in Table 4. Platelet function was assessed by both the PFA-100 and the LTA testing. PFA-100 results didn’t present any statistical significance between the control and the patient group (*p* = 0.50). When a more sensitive assay for detecting platelet function abnormalities [17] was performed, platelet aggregation was found to be defective in the PAH group in comparison to the control group (*p* < 0.001 in the LTA Epi testing and *p* = 0.004 in the LTA ADP testing). Apart from the decreased percentage of maximum platelet aggregation, the LTA ADP assay, also, showed that the formed platelet aggregates in the patient population were significantly less stable than those of the control group (59.1% of the PAH group showed disaggregation, *p* = 0.045).

ROTEM testing revealed that clot formation was found to be impaired in the PAH group compared to controls. More specifically, the initiation of the clotting process and clot propagation were shown to be defective in the patient population (CT: *p* = 0.02, CFT: *p* = 0.001 and anangle *p* < 0.001). The Li60 parameter was found to be decreased in the PAH group (*p* = 0.02), which is indicative of increased fibrinolysis. In contrast, clot firmness was comparable in both groups (MCF: *p* = 0.84).

ETP assay showed that the thrombin formation capacity of the PAH group was impaired in relation to the control group; 3 parameters in the patient population were found to be decreased compared to controls (AUC: *p* < 0.001, Cmax: *p* = 0.004 and Tmax: *p* = 0.008).

### 3.2. Markers of Platelet Activation and Endothelial Dysfunction

The results of the tests assessing markers of platelet activation and endothelial dysfunction are presented in Table 5. Markers of platelet activation (serotonin and TXA2) were found to be significantly increased in the PAH group when compared to controls (*p* = 0.002 and *p* = 0.03, respectively). The markers of endothelial dysfunction, VWF:Ac and VWF:Ag, were found to be decreased in the same population (*p* < 0.001), along with the vWF Ac/Ag ratio (*p* = 0.02). Lastly, p-selectin was found to be significantly elevated in the patient group when compared to controls (*p* = 0.03).

We performed a subgroup analysis to compare the test results of CTD-PAH and iPAH patient populations for all the laboratory methods used in this study. No statistically significant results were obtained.

## 4. Discussion

The results of the assays that evaluated hemostasis globally were able to validate the findings of our previous study [9]. PAH patients were shown to have: (1) diminished platelet aggregation, (2) unstable platelet aggregate formation, (3) impaired initiation of the clotting process and clot propagation, (4) defective thrombin formation capacity, (5) normal final clot firmness and (6) accelerated rate of clot lysis.

These findings portray a complex impairment of the hemostatic processes in the PAH setting. Primary hemostasis appears to be defective in that population, as shown by the decreased percentage of platelet aggregation and the presence of disaggregation in LTA testing. Secondary hemostasis is, also, impaired as suggested by the ROTEM results. The prolonged CT parameter of the ROTEM assay reflects the decreased rate of fibrin formation, which is dependent on thrombin formation [18]. The results of the ETP testing, where the total amount of free thrombin in the plasma, Cmax and Tmax were found to be decreased, support the CT finding.

The prolonged rate of initial clot formation, depicted by the CFT parameter of the ROTEM assay, can be attributed to low platelet count, poor platelet function or low fibrinogen [19]. Since our patient population had normal platelet counts and fibrinogen levels, this finding can be explained by a defective platelet function, which is supported by the results of the LTA testing. The diminished a-angle reflects the decreased rate of clot formation and is indicative of clot instability [18]. The MCF parameter is within normal ranges suggesting that even though platelet function is poor and clot propagation is defective, the final clot firmness (before fibrinolysis commences) is normal. Lastly, the decreased Li60 parameter is suggestive of increased fibrinolysis [19].

In order to explain the platelet function abnormalities that were observed in PAH patients in our previous paper, we hypothesized that platelets had already been activated before the time the tests were performed. In the present paper, we assessed serotonin and TXA2 serum levels as markers of platelet activation in order to test our hypothesis. Both substances were found to be increased, a finding consistent with this theory. It has been well established in the literature that serotonin levels are elevated in PAH patients [20]. This observation has led to the formulation of the “serotonin hypothesis of PAH”, where serotonin was thought to be a factor in the development of PAH, and, therefore a possible target for future therapeutic developments. In the serotonin hypothesis, the increased serotonin levels were associated with platelet storage pool defects [20]. In our study, we demonstrated not only that serotonin levels were increased in our patient population, but that they also exhibited platelet storage pool defects, as portrayed by the disaggregation findings [21].

TXA2 was also found to be increased in the PAH group of this study. Several studies have portrayed increased levels of thromboxane B2, an inactive metabolite of TXA2, in PAH patients and TXA2 receptor antagonists are being tested as potential treatments for PAH [22,23]. Disruption of the prostacyclin-thromboxane A2 pathway is one of the cornerstone processes that can contribute to the emergence of the pulmonary vascular defects observed in the PAH setting. The prostacyclin-thromboxane A2 pathway of PAH patients may have been shifted from the production of prostacyclin to the production of an alternative substance TXA2, which can lead to platelet aggregation, vasoconstriction and proliferation [24]. Increased levels of TXB2 levels have also been associated with worse World Health Organization functional class scores [25]. P-selectin, a marker of platelet activation and endothelial injury, was also found to be elevated in the patient group of this study. The evidence in the current literature regarding p-selectin appears to be contradictory with some studies demonstrating normal plasma levels of p-selectin in PAH patients, while others elevated [26].

Lastly, we evaluated VWF:Ag and VWF:Acin our study population, which were found to be decreased in PAH patients compared to controls, along with the VWF Ac/Ag ratio. Some studies in the field have linked PAH with acquired von Willebrand syndrome [27,28]. VWF multimers are particularly large in size, making them vulnerable to biophysical forces, including shear stress [29]. The increased shear stress of the pulmonary vasculature in PAH can lead to conformational changes of the VWF multimers, which can result in acquired von Willebrand syndrome and, therefore, in reduced VWF:Ac and vWF Ac/Ag ratio [29,30].

PAH is a chronic and progressive disorder, where endothelial dysfunction plays a central role in its development and evolution [27]. The chronic injury to the pulmonary endothelium could result in chronic platelet and coagulation cascade activation, extended platelet degranulation and, subsequently, in platelet function abnormalities [31]. The chronic activation of the patients’ procoagulant pathways could lead to the exhaustion of coagulation factors and, consequently, to their diminished hemostatic capacity [31,32,33]. Thus, our findings of decreased platelet aggregation, disaggregation, prolonged CT and CFT values, reduced thrombin generation capacity and increased levels of markers of platelet activation are consistent with that theory. The elevated D-dimer levels and the mild prolongation of the INR and aPTT parameters could also reflect the prolonged activation of the coagulation processes.

In the current literature, evidence of increased thrombin generation [34], as well as increased vWF levels in PAH patients [35], can be found. Contradictory evidence regarding fibrinolysis [7] and other substances, that could play an important role in the hemostatic deficits observed in the PAH setting (namely soluble thrombomodulin and plasminogen activator inhibitor-1) [36,37] have also been reported. The test results of this study showed decreased levels of thrombin generation and vWF, and increased fibrinolysis. These discrepancies could be attributed to differences in study designs, as well as factors such as patients’ PAH-specific therapies (some PAH-specific medications, e.g., prostacyclin analogues, can have discernible effects in hemostasis [1]), comorbidities and concurrent medications, the stage, duration and progression of their disease, as well as the different laboratory techniques used.

The MCF parameter was found to be within normal limits in our patient population. A low MCF is indicative of decreased platelet number or function, decreased fibrinogen level or fibrin polymerization disorders [38]. In our study, PAH patients demonstrated both platelet count and fibrinogen levels within normal ranges, and only platelet function was found to be moderately impaired. Thus, the mild platelet dysfunction may not be sufficient to affect MCF to a significant extent, and this is in keeping with the absence of bleeding events in these patients.

The main limitation of our study is its small sample size and, therefore, it should only be viewed as hypothesis-generating. Larger observational studies are required in order to firmly determine PAH patients’ coagulation profiles. Nevertheless, our results could help explain the contradictory reports in the literature of both thrombotic and bleeding phenomena observed in the PAH setting [39,40]. Coagulation can be described as a complex and dynamic equilibrium. In the case of PAH, the already activated and dysfunctional hemostatic cascade can abnormally shift towards thrombosis or hemorrhage depending on individual patient characteristics like, the presence of thrombophilic predisposition (which is often the case in PAH patients) or, on the contrary, of conditions that could increase patients’ bleeding risk (e.g., gastrointestinal disease, esophageal varices, liver or kidney dysfunction), or even by factors like the severity, trajectory and duration of the disease [41].

## 5. Conclusions

The results of this study suggest that the platelets of PAH patients are activated and present functional abnormalities. Hemostasis exhibits several other impairments as well, such asprolongation of the initiation of the clotting process and clot propagation, diminished thrombin generation capacity and alterations in VWF:Ac and VWF:Ag. These coagulation abnormalities suggest that the procoagulant activity of PAH patients may be impaired, probably due to a sustained and prolonged activation of the procoagulant processes and to chronic endothelial injury. These complex hemostatic abnormalities need to be further evaluated by well-designed clinical studies and assessed for potential clinical applications.

## Figures and Tables

**Table 1:** Measured parameters of rotational thromboelastometry (ROTEM).

Clotting Time (CT)	Time from the Start of Measurements Until the Formation of a Clot 2 mm in Amplitude
Clot formation time (CFT)	Time from the end of the CT until clot firmness of 20 mm is achieved
α angle (α°)	The angle between the central line (x axis) and the tangent of the TEM tracing at the amplitude point of 2 mm, describing the kinetics of clot formation
Maximum clot firmness (MCF)	It assesses the final clot firmness
Lysis index at 60 min (LI 60)	The percentage of the remaining clot stability in relation to the MCF following the 60 min observation period after CT, which describes the speed of fibrinolysis

**Table 2:** Measured parameters of thrombin generation assay.

**Area Under the Curve (AUC)**	Total amount of thrombin generated
**Lag Time (tlag)**	Time needed until the onset of thrombin generation
**Maximum Thrombin Concentration (Cmax)**	Peak thrombin generation
**Tmax**	Time needed to reach peak thrombin generation

**Table 3:** Demographics, clinical characteristics and laboratory findings of patients with pulmonary arterial hypertension (PAH) and healthy controls. Hemodynamics of PAH patients.

Add Title	Healthy Controls (*n* = 25)	PAH Patients (*n* = 44)	Significance
Age (years)	60.1 ± 10.2; 63 (52–67)	63.3 ± 9.8; 62 (56–71)	*p* = 0.22
Female (%)	16/25 (64%)	31/44 (70.5%)	*p* = 0.63
Caucasian Race (%)	25/25 (100%)	44/44 (100%)	*p* = 0.99
Patients (%) with a smoking history	7/25 (28%)	9/44 (20.5%)	*p* = 0.74
PLTs (10^3^/μL)	254 ± 47; 240 (228–292)	239 ± 65; 240 (195–266)	*p*= 0.30
WBC (/μL)	6938 ± 1186; 6880 (5900–7525)	7226 ± 1606; 7070 (6250–8355)	*p* = 0.45
Hb (g/dL)	13.3 ± 1.9; 13.2 (12–15)	13.9 ± 1.9; 14 (13–15)	*p* = 0.39
INR	0.97 ± 0.09; 0.96 (0.9–1.02)	1.03 ± 0.14; 1 (0.9–1)	*p* = 0.09
aPTT (sec)	29.8 ± 2.9; 29.1 (28–31)	32.3 ± 5.9; 32.3 (31–36)	*p* = 0.07
Creatinine (mg/dL)	0.85 ± 0.2; 0.8 (0.7–1.0)	0.92 ± 0.22; 1 (0.8–1)	*p* = 0.29
AST (U/L)	19.5 ± 12.7; 15 (11–22)	17.4 ± 6.5; 17 (12.5–20)	*p* = 0.39
ALT (U/L)	15.4 ± 6.9; 14 (9–21)	16.3 ± 8.6; 14 (12–18)	*p* = 0.68
Fibrinogen (mg/dL)	310.9± 88.8; 275 (217–343)	332.4 ± 102.7; 320 (247–408)	*p* = 0.14
D–dimers (ng/mL)	323.4 ± 125.5; 297 (267–359)	683.4 ± 439.3; 654 (353–828)	*p* < 0.01
mPAP (mm Hg)		41.2 ± 12.2; 40 (32–48)	
PVR (Wood units)		8.5 ± 3.3; 8 (6–10)	
CI (L/min/m^2^)		2.3 ± 0.5; 2.3 (1.9–2.5)	
NT–proBNP (pg/mL)		2676 ± 3526; 1615 (580–2787)	
6MWT (m)		329.9 ± 106.1; 315 (277–405)	

Abbreviations: aPTT, activated partial thromboplastin time; AST, aspartate aminotransferase; ALT, alanine transaminase; BMI, body mass index; CI, cardiac index; Hb, hemoglobin; INR, international normalized ratio; mPAP, mean pulmonary arterial pressure; NT-proBNP, N-terminal pro brain natriuretic peptide; PVR, pulmonary vascular resistance; PLT, platelets; WBC, white blood cell; 6MWT, 6 min walk test.Footnote: Data are presented as means ± standard deviations; medians and interquartile ranges in parentheses, or percentages when appropriate.

**Table 4:** Coagulation parameters in patients with pulmonary arterial hypertension and healthy controls.

	Healthy Controls (*n* = 25)	PAH Patients (*n* = 44)	Significance
CEPI CT (sec)	134 ± 14.8; 137 (123–145)	144.5 ± 75.7; 122 (97.5–169.3)	*p* = 0.50
LTA Epi (%)	70.8 ± 16.1; 68 (59–84)	51 ± 22; 57.5 (32.8–68.75)	*P* < 0.001
LTA ADP (%)	73.1 ± 11.1; 71 (64––78)	58.9 ± 16.7; 61.5 (46.5–70)	*p* = 0.004
Patients (%) with disaggregation	0/25 (0%)	26/44 (59.1%)	*p* = 0.045
CT (sec)	481.4 ± 116.5; 475 (409–562)	732.3 ± 407.4; 704 (448–914)	*p* = 0.02
CFT (sec)	133.1 ± 29; 125 (113–158)	390.1 ± 331.8; 309 (217–496)	*p* = 0.001
α angle (α°)	63.9 ± 5.4; 65 (56–61)	48.6 ± 15.6; 51 (38–59)	*p* < 0.001
MCF (mm)	59 ± 4.1; 58 (56–61)	59.5 ± 14.9; 60 (52–68.5)	*p* = 0.84
Li60 (%)	94.2 ± 2.8; 95 (91–97)	92 ± 6.1; 93 (90–97)	*p* = 0.02
Lag time (sec)	30.2 ± 5.1; 28.9 (26.8–32)	28.1 ± 6.9; 27.1 (25.6–30.7)	*p* = 0.24
Tmax (sec)	82.8 ± 16.7; 79.3 (69.2–89.3)	60.9 ± 12.7; 60 (53–65)	*p* < 0.001
Cmax (%)	111.3 ± 14.1; 110 (101–119)	97.1 ± 21.1; 100 (91–110)	*p* = 0.004
AUC (%)	101.1 ± 11.9; 98 (93–110)	84.7 ± 28.5; 87 (76–99)	*p* = 0.008

**Table 5:** Markers of platelet and endothelial dysfunction in patients with pulmonary arterial hypertension and healthy controls.

	Healthy Controls (*n* = 25)	PAH Patients (*n* = 44)	Significance
Serotonin (ng/mL)	213 ± 127.9; 203.3 (114.6–297.5)	1064.9 ± 1125.9; 836.7 (268.5–1265.9)	*p* = 0.002
Thromboxane A2 (pg/mL)	104 ± 37.2; 102.4 (83.5–125.2)	265.7 ± 341.2; 121.6 (96.6–136.4)	*p* = 0.03
Soluble p–selectin (pg/mL)	2018.1 ± 558.8; 1964.4 (1696.7–2371.8)	2341.9 ± 346; 2291.5 (2165.6–2517.4)	*p* = 0.03
vW:Ac (%)	137.5± 27.9; 149.8 (126.7–158)	92.2 ± 29.4; 89.9 (71.7–109.7)	*p* < 0.001
vW Ag (%)	180.5 ± 46.4; 184.2 (149.3–217.7)	105.8 ± 26.5; 100.6 (90.3–119.8)	*p* < 0.001
vW Ac/Ag	0.86 ± 0.15; 0.89 (0.8–0.94)	0.77 ± 0.12; 0.77 (0.68–0.86)	*p* = 0.02

Footnote: Data are presented as means ± standard deviations; medians and interquartile ranges in parentheses, or percentages when appropriate.
